# Gaps in environmental and social evidence base are holding back strategic action on our national food system

**DOI:** 10.1080/03036758.2025.2526838

**Published:** 2025-07-03

**Authors:** Nick W. Smith, Richard W. McDowell, Conal Smith, Meika Foster, Charles Eason, Miriana Stephens, Warren C. McNabb

**Affiliations:** a Sustainable Nutrition Initiative, Riddet Institute, Massey University, Palmerston North, New Zealand; b AgResearch Ltd, Lincoln Science Centre, Lincoln, New Zealand; c Faculty of Agriculture and Life Sciences, Lincoln University, Lincoln, New Zealand; d Kōtātā Insight, Wellington, New Zealand; e AuOra Ltd, Wakatū Incorporation, Nelson, New Zealand; f Edible Research Ltd, Ohoka, New Zealand

**Keywords:** Food systems, sustainability, food strategy, agricultural policy, food policy

## Abstract

While there is broad agreement on the challenges facing the Aotearoa New Zealand food system now and in the near future, there is less agreement on the action to be taken. Poor agreement is fuelled by gaps in both our scientific understanding of the food system and data to support our decision making, particularly in the environmental and social spaces. Filling these gaps and being transparent about scientific confidence in future predictions will strengthen the evidence base for action.

The Aotearoa New Zealand (Aotearoa-NZ) food system faces many recognised challenges, such as: a large environmental footprint, vulnerability to natural and market shocks, and worsening population nutritional health. Several high-level publications from government, the food industry, and specialist thinktanks have been produced in recent years documenting the need for change and outlining potential pathways for change (Jones et al. 2019). However, confidence in these pathways is limited due to (a) a lack of robust sustainability data beyond the major, national-level indicators (e.g. emissions, GDP) and established sectors, and (b) a lack of systems to test if actions taken today will have the desired outcome in the future.

Kai Anamata Mō Aotearoa – a five year research programme exploring scenarios for the New Zealand food system, co-led by the Riddet Institute and Wakatū Incorporation – conducted a series of workshops involving food system stakeholders with national-level interests, aiming to understand the challenges these stakeholders see to our food system, the questions they want answered about the future of the food system, and what information they would need to make confident decisions on future action. These took place in July 2024 in Wellington and Auckland, with brief introductory context talks followed by facilitated table discussions and follow-up surveys. Attendees included representatives from central and local government, primary industries and adjacent bodies, Māori organisations, food charities, environmental groups, and research institutions.

## Challenges identified by food system stakeholders

Consensus on the major challenges facing our food system was broad and unsurprising: Te Taiao (our natural environment), human health and capital, economic vulnerability, and the lack of a strategic food system vision. The specifics varied, with emerging, less widely discussed challenges appearing, but these major topics appeared in almost every discussion.

The two-way relationship between our food production and Te Taiao, and the unacceptable current state of this relationship, were discussed at every table. Climate change and water were the most discussed topics, with concern about our ability to deal with both the gradual and acute impacts of climate, and the effects of climate and land use on the quality, access, availability, and ownership of water.

The relationship between food and population health was another major concern at most tables, although we maintain that food production's relationship with the environment is more direct than its connection with consumption and health. Poor food security, access, and knowledge were related challenges discussed, with serious consequences for human wellbeing and productivity extending intergenerationally. People’s relationship with agriculture in this country was also highlighted at both workshops: its unattractiveness as a career, and challenges to social license, trust, and reputation were all recurring topics.

Economically, our export-oriented production system, while producing high market returns, was also identified as a major vulnerability by multiple tables. Several examples were raised of upcoming regulations from overseas customers (particularly environmental regulations or accreditation schemes) that outpace our domestic regulations, threatening market access if we fall short of increasingly adopted international targets (e.g. Science Based Targets Network 2025).

Finally, attendees at both workshops complained of the lack of a strategic, long-term vision for our national food system, despite many calls for formalising shared priorities to guide policy and action (Riddet Institute 2012; The Aotearoa Circle 2022; Our Land and Water 2023). Some felt that action was needed to move towards a ‘feeding Aotearoa-NZ first’ mentality, but it was questioned by others how this could feasibly be implemented against the significant challenge of a food system driven by export returns.

While the topics identified here have been long discussed in fora across the country, one key issue highlighted in our workshops was the lack of good metrics for all aspects of the system to indicate movement in the right direction. Lack of measurement means a lack of evidence to support change, and translating overseas numbers or approaches will not always be applicable here.

## Investigating the implications of change

We also asked attendees to imagine that they had a perfect replica of the national food system, and to tell us what changes they would experiment with, and what parts of the system would signal whether changes were positive.

The most prevalent discussions related to land use or production. Land use diversification or relocation, changing management practice, or replacing one land use with another were of great interest, as was the idea of increased localisation of food production with consumption. We note that many of the land use change questions are the subject of significant existing work, particularly as part of the Our Land and Water National Science Challenge, which developed several models for land use change at different scales to achieve various environmental and economic goals. However, gaps remain both in data availability and uptake of this research.

Also popular were scenarios testing the impact of specific policy changes (e.g. regulatory, trade, and property) to systematically explore their consequences. Some attendees were interested in exploring the resilience of Aotearoa-NZ’s food system to external shocks, while others were focused on different ways to manage environmental outcomes or increase economic returns.

We provided two ways for attendees to indicate useful measures for tracking food system change. The first was to rank the importance of a selection of measures already being studied (see Table 1), and the second was to propose new, additional measures with strong potential.

**Table 1.** Attendees (n = 22) were asked to rank the below measures for their importance in food system decision-making, from 1 ‘this is never important in my decisions’ to 7 ‘I always consider this in my decisions’. IQR: interquartile range; SD: standard devitation.

Outcome	Median ranking (IQR)	Mean ranking (SD)
Water quality	6.5 (2)	5.91 (1.24)
Nutritional content of food produced	6.5 (2)	5.73 (1.68)
Greenhouse gas emissions	6 (1.75)	6.05 (0.98)
Soil erosion	6 (2)	5.68 (1.49)
Carbon sequestered	6 (1.75)	5.50 (1.12)
Employee wellbeing	6 (2)	5.36 (1.8)
Workforce health outcomes	6 (2.75)	5.36 (1.85)
Total economic return to enterprise	6 (3)	5.18 (1.99)
Workforce skills and qualifications	6 (3)	4.95 (2.2)
Biodiversity	5.5 (2)	5.32 (1.14)
Employee job satisfaction	5.5 (2.75)	4.50 (2.15)
Energy use	5 (1.75)	5.14 (1.39)
Operational costs	5 (2.75)	5.14 (1.6)
Export value of products produced	5 (3)	5.09 (2.04)
Capital investment for land use change	5 (3.75)	4.95 (1.97)
Profit per hectare	5 (3.5)	4.73 (1.93)
Government subsidy expenditure	5 (3.75)	4.55 (2.19)
Median wage	5 (3)	4.50 (2.02)
Industry regulatory burden	5 (3)	4.50 (2.02)
Mass of products produced	5 (1)	4.36 (1.55)
Total irrigation water used	4.5 (2)	4.86 (1.29)
Total fertiliser use	4.5 (2)	4.82 (1.47)
Crop yield	4.5 (1)	4.55 (1.44)
Government expenditure on compliance monitoring	4.5 (1.75)	4.45 (1.53)
Hectares of land used	4 (1.75)	4.23 (1.54)
Workplace injuries	4 (2.75)	4.18 (2.04)
Number of FTE	3.5 (2.75)	4.00 (2.13)
Worker loneliness	3.5 (2.75)	3.68 (1.84)
Capital depreciation per hectare	3.5 (2)	3.23 (1.7)
Number of seasonal workers	3 (4.5)	3.36 (2.21)

In the former exercise, environmental measures (including greenhouse gas emissions, water quality, soil erosion, and carbon sequestration) scored highest for importance, along with the nutritional content of food, human health and wellbeing measures, total economic return and workforce skills (Table 1). The latter discussion of additional measures was dominated by food security, nutrition, and employment topics. For food and nutrition measures there was less agreement on the best ways to quantify improvement, whereas job security, farmer capability, labour demand, unemployment, wages, and household incomes were all mentioned on the employment side.

Several outcomes mentioned by attendees related to indigenous aspects of the food system and indicators of cultural wellbeing, some of which are more quantifiable than others. These included the extent of Mahinga Kai practice, degree of Iwi and Hapū leadership in the system, Māori food sovereignty, and the status of Taonga species. A great diversity of less tangible social outcomes was also discussed: equity (of food access, measurement, wealth), identity, resilience, and public perception of agriculture.

## Implications for food system planning

Calls for a strategic vision, or specifically for a National Food Strategy, have been widely made over the last several years, and were echoed here. The workshops showed the importance of being able to quantify outcomes cited in any strategy. Measurement provides an evidence base that can be used in modelling and planning, and predicting performance of future strategies. There are several examples of countries that have developed national food strategies drawing on system modelling, including Ireland, the UK, and Finland, recognising the value of this approach. Aotearoa-NZ must achieve government buy-in to the value of this strategic approach.

Some environmental measures are well defined and lend themselves to future projection; others are more challenging. The carbon balance of a transition from sheep farming into pine forestry is easily converted into a single, nationally-representative number, whereas its impact on catchment water quality depends on farm management, the interaction of management with catchment characteristics, and a diverse array of other locally-dependent processes. While the spatial arrangement of land use can be modelled and altered to achieve the best possible outcomes for water quality or carbon emissions (McDowell et al. 2022), other issues are yet more challenging to consider, such as judging the value of local biodiversity in one location or jurisdiction versus another. This is an area where primary data collection and modelling is underdeveloped in Aotearoa-NZ, despite its popularity in the workshop discussions.

The impacts of changes to the food system on human wellbeing are clearly important, and received strong recognition in the workshops. Data exists to tie various indicators of human wellbeing to the food system in Statistics New Zealand’s Integrated Data Infrastructure (IDI) by leveraging information on geography, occupation, and industry. This would allow a better understanding of how changes in land use and food sector employment impact on a wide range of economic and social outcomes ranging from wage rates through to work place injuries, job satisfaction, and overall life satisfaction. Thanks to the IDI, Aotearoa-NZ is well placed to take the lead globally in measuring and modelling the connections between the food system, land use change, and measures of human wellbeing.

Population nutrition, while highly important, is indirectly linked to food production via complex causal chains, and thus challenging to link in future planning and modelling. The nutritional content of food produced and traded is easily calculated, but food access and consumption, food security, and health outcomes are strongly driven by behavioural, microeconomic, and social factors, challenging their inclusion in production or international trade modelling. While measuring current state and quantifying the benefits of change to population nutrition is possible (Cleghorn et al. 2022), achieving the necessary change has proven much harder and should remain a focus topic in public health.

Capturing the level of confidence in different data and future predictions reliant on this data will be essential for food system modelling. There is a great difference between our confidence in modelling the labour units needed to support future expansion of an existing industry, compared to modelling the future average wage in an emerging industry, or the implications of adopting novel, alternative land uses. Clear, concise confidence ratings of outputs and outcomes (e.g. high, medium, or low confidence, akin to the IPCC approach (Mastrandrea et al. 2011)) should become standard in reporting calculated values.

There is also a need to consider transition variables. What is the time necessary to achieve a future change to the food system? What capital, skills, and knowledge are also needed in this transition? When are the costs incurred and the benefits felt? Planting indigenous forest today incurs immediate costs and benefits, with other costs and benefits felt over decades, and the full scope of impacts realised over centuries. We call on the sector community to consider how such transition thinking fits with their current approaches.

If the desired outcome is that we move from an export-oriented food system to one that considers the needs of New Zealanders before those overseas (as was expressed by many workshop attendees), we need to have data to support the viability of this change. At present, no framework exists that captures flows of agricultural and food commodities within Aotearoa-NZ, which makes quantifying the dependence on food supply from outside a region challenging. However, the qualities of local food systems have sparked detailed study internationally (Nemes et al. 2023), indicating that modelling within the context of Aotearoa-NZ is a meaningful goal.

To summarise, we believe food system change must be built on a transparent evidence base and rigorously tested via tools such as modelling. The evidence base and modelling is seen as strong for some measures, but weak in others, particularly the social consequences of change and certain environmental outcomes. Quantifying our confidence in data and predictions in an accessible way and clearly communicating the transition aspects of changes are key for transparency. Addressing these issues would remove some of the uncertainty driving different proposed strategies to countering food system challenges.

## Acknowledgements

The *Kai Anamata mō Aotearoa: exploring future food system scenarios and impacts* Endeavour Research Programme is funded by the New Zealand Ministry of Business, Innovation and Employment (Contract MAUX2305). The authors would like to thank the wider research team for their contributions, and the workshop attendees and their organisations for their time.
